# Response Activity in Mixed-Method Survey Data Collection—The Methods Used in a Survey among the Foreign-Born Population in Finland (FinMonik)

**DOI:** 10.3390/ijerph18063300

**Published:** 2021-03-23

**Authors:** Hannamaria Kuusio, Anna Seppänen, Laura Somersalo, Satu Jokela, Anu E Castaneda, Rekar Abdulhamed, Eero Lilja

**Affiliations:** Finnish Institute for Health and Welfare, 00271 Helsinki, Finland; anna.seppanen@thl.fi (A.S.); laura.somersalo@thl.fi (L.S.); satu.jokela@thl.fi (S.J.); anu.castaneda@thl.fi (A.EC.); rekar.abdulhamed@thl.fi (R.A.); eero.lilja@thl.fi (E.L.)

**Keywords:** migrants, health, well-being, access to care, integration, epidemiology, health policy

## Abstract

In terms of the number of respondents, Survey on Well-Being among Foreign Born Population (FinMonik) is so far the most extensive survey carried out among the population with foreign background in Finland. It comprises a wide range of self-reported data, including information on the respondent’s health, well-being and access to care, which can be widely utilized in planning and assessing integration, health and welfare policies. A mixed-method approach (an electronic questionnaire, a paper questionnaire and phone interviews) was used in collecting the data which consists of responses by 6836 respondents aged 18–64 years. All response types included, the response rate was 53.1% (*n* = 6836). This study describes in detail the methods used in the FinMonik survey. In addition, we describe the demographics of the respondents partaking in each response format. The aim of the study is to promote the development of mixed-method survey as a way of collecting reliable data that can be used to enhance foreign-born people’s health, well-being and access to health care. The survey responses will be used as a baseline in observing the respondents’ well-being through the register-based data available in several national registers on health, medicine use and access to care as well as the data collected in the study Impact of Coronavirus Epidemic on Well-Being among Foreign Born Population Study (MigCOVID). Furthermore, the FinMonik study protocol will be repeated every four years.

## 1. Introduction

The UN estimated that 272 million people currently live outside their country of birth, the number accounting for about 3.5% of the world’s population [1]. Approximately 82 million of these people live in Europe. In Finland, the number of migrants has doubled in the last ten years, now accounting for 7.3% of the country’s total population [2]. Previous studies across Europe have shown that there exists inequality in health and well-being as well as access to health care [3,4,5,6] between foreign-born and the general population. Similar results have also been obtained in Finnish population studies [7,8]. Migration itself has not been recognized as a risk for health and well-being, but the conditions surrounding the migration process seem to increase vulnerability to health problems, particularly among those who migrate involuntarily.

In order to reduce health inequalities, genuinely representative information about migrants’ health, well-being and access to care is needed. In Finland, a population-based survey consisting of health examination and interview was conducted among persons of Russian, Somali and Kurdish origin in 2012 [6]. The study was followed by the Survey on Work and Well-Being among People of Foreign Origin (UTH) [7]. Both studies succeeded in collecting reliable information on migrants’ health and well-being. However, regular and systematic population-based surveys have not been conducted among the migrant population in Finland. Regular and systematic data collection, using the same methods, target group and indicators, while ensuring comparability across all population groups, is essential when the aim is to reduce health-related inequality experienced by migrants [9].

A questionnaire survey using a random sample of the population provides important data that can be used in public health research, epidemiology and policy making [10]. It is valuable to obtain a large number of participants relatively cost-effectively while maintaining a full geographic coverage of the target population [11]. Participation rate is an important factor in representativeness, and it often causes problems in randomized surveys. The downward trend in response activity has been a universal concern for decades [12,13,14]. Previous studies have identified several methods that can be used to increase the number of responses in randomized survey questionnaires [15,16,17,18]. A teaser (e.g., a pen) inside the envelope and a financial incentive on condition of response more than doubled the response rate, while the response rate almost doubled when incentives were not conditional on response. Addressing respondents personally can also enhance the response rate. Moreover, short questionnaires are more probable to be responded to than long ones, and those who are contacted by phone or letter before sending the questionnaire are more likely to respond. Furthermore, combining an Internet questionnaire with a paper follow-up questionnaire and phone interviews (mixed-method approach) could increase the response activity [19]. Previous Finnish population studies have utilized culturally sensitive methods in the data collection process, such as recruiting personnel with foreign background, using multilingual study material and training fieldwork staff to use standard protocols and questions to ensure comparability between the study groups [20,21].

Survey on Well-Being among Foreign Born Population (FinMonik) used mixed methods such as electronic questionnaires, paper questionnaires and telephone interviews in gathering data. The main objective of the FinMonik survey was to collect reliable data on health, well-being and service use among people with foreign background born outside Finland and currently living in Finland (PFB). The aim was to obtain respondents living in all regions of mainland Finland. Furthermore, the survey was used for piloting electronic questionnaire as a method of data collection to see whether it could be used instead of more costly and time-consuming face-to-face interviews to gather reliable data. The information provided by the FinMonik survey is used to monitor integration, health, well-being and access to care among PFB nationally and within the 18 Finnish counties as well as the six largest cities in Finland. In addition, the data are and will be widely used for research purposes. The study protocol will be repeated every four years. Furthermore, the well-being of the participants in the baseline will be observed using register-based data available in several national registers on health, medicine use and access to care. The register data provides comprehensive information on the health status of those persons who have used health services in Finland. The survey data can be used to assess, for example, the need for services and treatment in services or perceived well-being.

Those who responded to the FinMonik survey will also be contacted in relation to the Impact of Coronavirus Epidemic on Well-Being among Foreign Born Population Study (MigCOVID) survey which aims to examine the effect of the coronavirus epidemic and the related restrictive measures on the daily life, health, quality of life and mood as well as the experiences of service use among those who responded to the FinMonik survey.

The aim of this paper is to present the methods of Survey on Well-Being among Foreign Born Population (FinMonik). In addition, we describe the demographics of the respondents partaking in each response format (the electronic questionnaire, the paper questionnaire and the phone interview).

## 2. Materials and Methods

**Figure 1:**
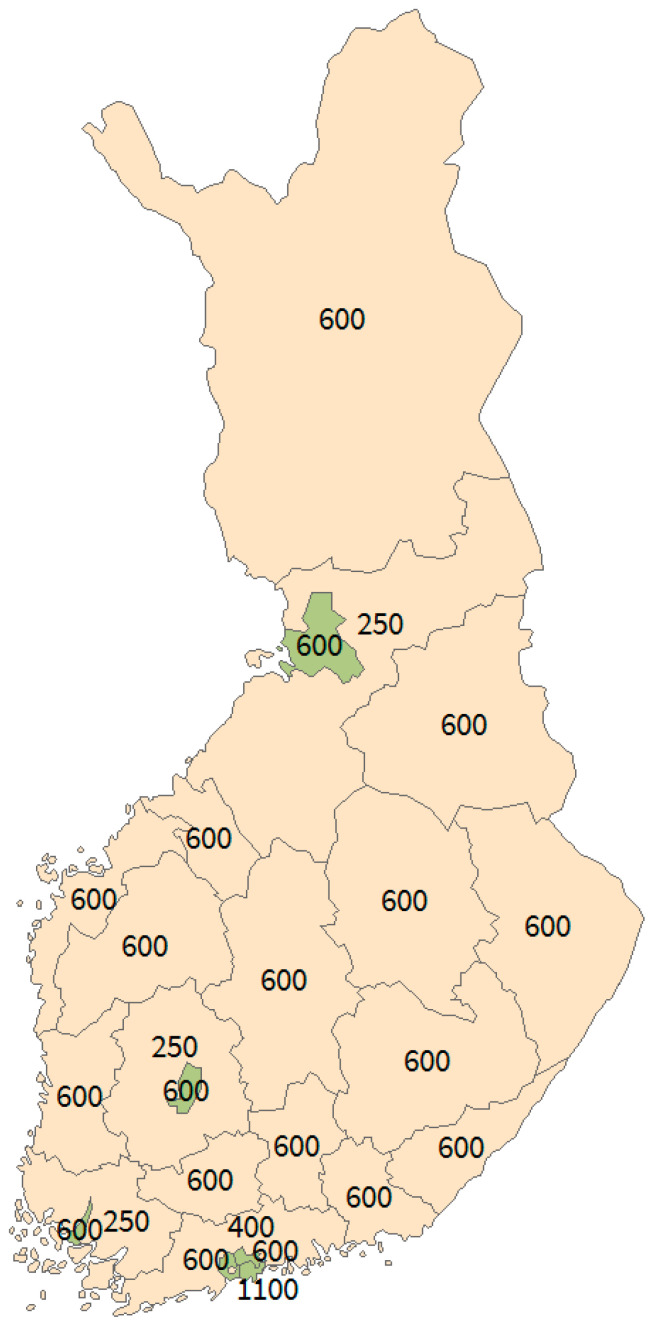
Map of mainland Finland including stratified counties and cities.

The sample of the FinMonik survey was based on a stratified random sampling, in which mainland Finland (Finland excluding Ahvenanmaa) was divided into 24 strata (Figure 1). All counties (*n* = 18) and six largest cities (Helsinki, Espoo, Tampere, Vantaa, Oulu and Turku) each formed its own stratum. At least 600 PFB were selected from each of the counties and cities. In the counties in which the central city formed its own partition (Uusimaa, Southwest Finland, Pirkanmaa, North Ostrobothnia), the sample size was set at 250 for the rest of the county. In Helsinki and Uusimaa, the sample size was increased to correspond a minimum of 1% of the target population (Uusimaa *n* = 400; Helsinki *n* = 1100). The over-coverage of the sample was estimated to be not more than 20%. Of the remaining sample, 50% were expected to participate, resulting in at least 250 respondents for each region. A sample of 250 was then estimated to lead to 95% confidence intervals of roughly ±6% for most regions when the point estimate was 50% and finite population correction and analysis weights had been taken into account.

The target population of the study consisted of persons of working age (18–64 years) with foreign background, who were born abroad and currently living in Finland (PFB). The sample was obtained in March 2018 from the population register maintained by the Digital and Population Data Service Agency. The sample was drawn using the following criteria: (1) the respondent’s country of birth must be other than Finland; (2) both parents or the only known parent of the respondent must have been born abroad; (3) the respondent must have lived in Finland for at least a year at the time of sampling; (4) the respondent must be aged 18–64 at the time of sampling, and (5) the respondent must not have come to Finland through adoption.

According to the Statistics Finland a total of 265,752 PFB live in Finland [2]. Of those, 13,650 individuals formed the sample and received the invitation letter to participate in the study. Those who received the invitation letter but had moved abroad after sampling or whose invitation letters was returned undelivered by the post were interpreted as over-coverage. The over-coverage proved to be lower than expected (5.7%; *n* = 773). Thus, after removing over-coverage (775 individuals) of the sample, the final sample consisted of 12,877 respondents.

### 2.1. Questionnaire

The questionnaire contained 78 topics related to different areas of life, including quality of life, welfare, participation in social and societal activities, experiences of discrimination, safety, self-perceived health, employment and competence (Table 1). Data were also collected about the need for and use of social and healthcare services and services related to employment and immigration as well as trust in these services. A short version of the questionnaire was produced to be used in telephone interviews, and it included around one fourth of the contents of the full questionnaire (Table 1). Only the most important indicators for monitoring integration were selected for the short version of the questionnaire. The number of questions was moderated so that the questions remained representative. The comparativeness to the general population was deemed essential in promoting equality between the different population groups. To make comparisons to the general population possible, most of the questions used were questions that had already been used in a similar study among the Finnish general population [22].

### 2.2. Study Population and Respondents

The invitation letter and the questionnaire were translated into 17 languages (from Finnish to Albanian, Arabic, Dari, Farsi, English, Spanish, Mandarin Chinese, Kurdish (Sorani), Polish, French, Swedish, Somali, Thai, Turkish, Russian, Vietnamese and Estonian), which meant that 77.1% of those invited to the study received the material in their declared mother tongue (later: mother tongue). The 16 most common languages in Finland were selected alongside with French, which was presumed to be common as a second language among the participants. Both invitation letter and questionnaire were translated using one service provider. The materials were reviewed by another service provider as well as the multilingual field personnel and interviewers, some of which had also been involved in previous population studies conducted by the Finnish Institute for Health and Welfare. Despite the reviewing process, some corrections were needed in later stages: the Somali translations were rejected entirely and subsequently re-translated, and some corrections were also made to the Kurdish translation before sending the second paper questionnaire. Responses obtained with the rejected translation were removed from the final data.

### 2.3. Data Collection Methods

**Figure 2:**
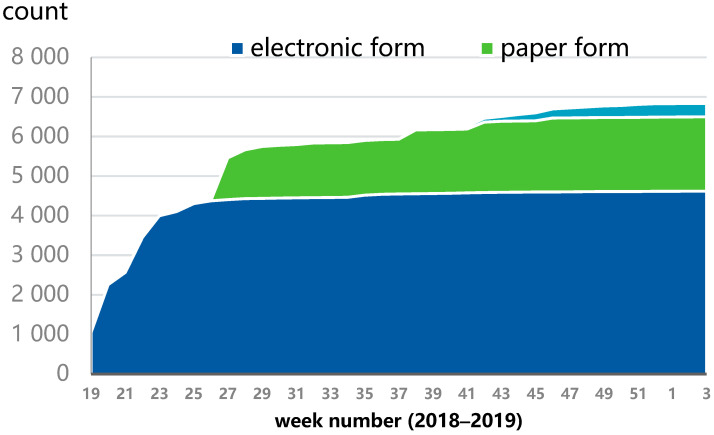
Data collection methods and number of respondents per week.

The data were collected between 7 March 2018 and 15 January 2019 primarily online with an electronic questionnaire, and it was supplemented with a paper questionnaire and telephone interviews and home visits in the city of Espoo (Figure 2).

In phase one (week 1, 7 May 2018), an invitation letter containing the web address of the electronic questionnaire as well as the personal ID and password was sent to the respondents’ home addresses. The letter was provided in one of the two official languages of Finland (Finnish or Swedish) and in the respondent’s mother tongue or in English if the questionnaire was not available in the respondent’s mother tongue. The invitation letter also described the purpose and content of the study, explained the protocols related to data protection and announced the chance to participate in a prize draw by answering the survey. A reminder text message in Finnish, in Swedish and in English was sent to those who did not fill in the questionnaire within ten days and whose telephone number could be retrieved (week 3; 18 May 2018). The phone number was retrievable through a phone number search service in case of a third of the sample. A reminder letter was posted to all non-respondents three weeks after the first invitation (week 4; 28 May 2018).

In phase two, six weeks after the first contact (15 June 2018), the paper questionnaire was sent to those who had not replied to the questionnaire over the Internet. Like the invitation letter, the paper form and its accompanying material were also provided in two languages. The second and last reminder was sent to non-respondents by post three months after the first contact (22 August 2018).

Telephone interviews started on 27 September 2018. The main aim of the telephone interviews was to encourage the participants to respond to either the electronic or the paper questionnaire. In addition, the opportunity to participate in an interview by telephone was offered, either immediately or at a time more convenient for the respondent. Computer-assisted telephone interview technique (CATI) was used to conduct the phone interviews. The respondents speaking the languages with the highest proportion in the sample or the languages with the lowest response rate thus far were selected to be interviewed by telephone. A total of nine multilingual interviewers acted as telephone interviewers, and the interviews were conducted in ten languages (Dari, English, Estonian, Farsi, Finnish, Kurdish (Sorani), Mandarin Chinese, Somali, Turkish and Russian). In addition to these languages, Arabic and Pashto and the Kurmanji dialect of Kurdish were used when needed.

Door-to-door visits were conducted in November 2018 in the city of Espoo. The purpose of these visits was to increase the number of participants in the area and to make the FinMonik survey more visible.

The door-to-door visits were carried out on five different days in November 2018. Before visits, a bulletin was sent that informed the subjects of a visit and listed the research team’s contact details. A total of 72 visits took place, but only 15 individuals of the study sample were reached this way. Seven of them refused to participate in the study, and eight people agreed to contact the telephone interviewer at a more convenient time. No interviews were conducted at the door. The visits did not significantly increase the number of participants, but they helped to make the survey known among the target population.

### 2.4. Ethical Consideration

The FinMonik study was granted an ethical approval by the Institutional Review Board (IRB) of The Finnish Institute for Health and Welfare (THL) (Decision number: THL/271/6.02.01/2018 §783). The invitation letter to the participants emphasized volunteering, protection of personal data and confidentiality. The invitation letter emphasized also that participation or refusal in the study did not affect their rights to Finnish health services.

Consent was obtained from the respondents to both participate in the survey (filled questionnaire) and to link the survey data to available register data. Respondents were assured they could stop and dropout anytime during the study.

## 3. Results

### 3.1. Respondents

A total of 6836 people participated in the study (Table 2). Of them, 35.9% (*n* = 4618) participated in the electronic questionnaire, 14.6% (*n* = 1878) to the paper questionnaire and 2.6% (*n* = 340) to the telephone interview. All response methods included, the response rate was 53.1%. The response activity varied between regions (46–60%). The response rates were the highest in Central Finland and Northern Savonia (60%). The response rates were the lowest in Southern Ostrobothnia and Southwest Finland (46% and 48%, respectively).

More women responded than men (56% vs. 50%) (Table 1). However, in the electronic questionnaire, the response rates of men and women did not differ (35% and 36%, respectively). The response rate varied with the age of the respondent: the highest participation was among those aged 55–64 years and the lowest among those aged 18–24 years (55% vs. 48%). Those aged 55–64 years responded to the electronic questionnaire somewhat less often (30%) compared to the younger age groups (45–54 years: 33% and 35–44 years: 33%). In the youngest age group (18–24 years), the response rate online was 38%, while among those aged 25–34 it was 39%. Those who were married or in a registered partnership participated in the study more often than those who were unmarried, divorced or widowed (58% vs. 46–53%). Those who were either single or married answered online more often than the widows and the divorced (38% and 39% vs. 23% and 25%).

The entrepreneurs and upper-level employees participated the most frequently (57%), while only 50% of the socioeconomically inactive participated. The entrepreneurs and upper-level employees and students answered online more often than the other employed and the unemployed or retired (41% and 40% vs. 34% and 32%). The respondents with higher education participated more often than those who had only received upper secondary education or less (62% vs. 50%). Those with a higher education degree answered online more often than the respondents who had received only primary- or secondary-level education (46% vs. 34% and 31%) also.

When it came to the country groups, the respondents in Asia or Middle East and North Africa groups (58% and 56%, respectively) participated in the survey more often than those in other country groups. The country groups with the lowest response rates were sub-Saharan Africa (46%) and Latin America, former Yugoslavia or other regions (45%). However, the lowest response rates for the online questionnaire were among those in the Estonia group (29%), while in Asia and the EU- and EFTA countries and the North America groups, the response rate for the online questionnaire was over 40%.

The paper questionnaire was the most popular form among the 55–64-year-old respondent and the widows (both 22%). Those who had lived in the country for less than ten years responded more often online than to the paper questionnaire. Those who had lived in Finland for more ten years responded slightly more often to the paper questionnaire than to the online questionnaire.

A short version of the questionnaire was used mainly in phone interviews. However, seven of those who participated in phone interviews were willing to fill a whole questionnaire. Thus, a total of 347 (2.7%) responded to a short questionnaire (not shown in the Table 2). Most respondents (*n* = 6271; 97%) answered all questions of the questionnaire he/she completed.

### 3.2. Combining Register-Based Data

The FinMonik data was combined with several national registers (Table 3). The aim of the combining was (1) to conduct non-response analyses to investigate how the respondents and non-respondents in the study differ from each other and how the non-response affects the result, (2) to compare survey-based and register-based information to verify and correct the data and (3) to supplement the information collected in the survey, e.g., to determine socioeconomic status, family situation, immigration background, health status, service use and employment situation.

### 3.3. Statistical Analyses

The respondents were categorized into seven country groups (Table 4). The categorization based on the country groups used in the Survey on Work and Well-Being among People of Foreign Origin (UTH) Study [7]. The category was determined by the person’s country of birth, except for those born in the former Soviet Union and the former Yugoslavia: Those who were born in Russia or the former Soviet Union and whose mother tongue (or citizenship) was Estonian were categorized as belonging to the Estonian group. Those born in Russia or the former Soviet Union whose mother tongue (or citizenship) was Latvian or Lithuanian were categorized as belonging to the EU, EFTA and North American group. Of the respondents born in the former Yugoslavia, those with a Slovenian or Croatian citizenship or with Slovenian or Croatian as their mother tongue were also categorized as belonging to the EU, EFTA and North American group.

Analysis weights were calculated to take into account the unequal sampling probabilities and the non-response. The response propensity-based weights were first calculated using the random forest method [25]. In the modelling, we used the available register information on age, sex, Finnish citizenship, country group, region of residence, marital status, age at migration, years lived in Finland, number of persons in the household, number of children in the household, availability of phone number, education and socioeconomic status. The propensity-based weights were further calibrated to match the distributions of the entire target population [26]. The calibration variables used were age, sex, stratum (area of residence), country group and education. The resulting analysis weights were then applied in all the statistical analyses. The weights were produced using packages random forest and icarus in R [27].

Many of the measures included in the questionnaire were dependent on age and sex. Since the distributions of age and sex differed between the countries of origin and from the general population model, adjustment was used when reporting the results. All results were adjusted for age using predictive margins [28]. In addition, the results were adjusted for sex when reporting the combined results for both men and women. The results were reported as percentages/mean values and their 95% confidence intervals, applying the analysis weights and the finite population correction with the stratification of the sample taken into account [29].

### 3.4. Data on the General Population

The FinSote National Survey of Health, Well-Being and Service Use [22] data were used for comparing the FinMonik survey population with the Finnish general population. The FinSote 2018 data were collected in 2017 and 2018 using a questionnaire that could be answered either online or by post. A stratified random sample of 59,440 was drawn from the population aged 20+, with 3300 people selected from each county. The overall response rate for the FinSote 2018 survey was 43%.

## 4. Discussion

In this study, we have described the methods used in the Survey on Well-Being among Foreign Born Population (FinMonik). We have also reported the demographics of the participants in each of the response formats (the electronic questionnaire, the paper questionnaire and the phone interviews). The generalizability and reliability of study results were affected by the research methods used, by the sample and the sampling method and by the participatory activity [9]. The key to success lies in the selection of participants. It is especially important to consider how the respondents may differ from those who, for one reason or another, have not responded to the survey. The FinMonik survey is based on a stratified random sample and its overall response rate is reasonably high (53.1%). The response activity is difficult to compare with other similar studies because the target group, size of the study population and data collection methods differ greatly. The response activity is usually higher (50–90%) if the migrant studies have been targeted to the population of a few countries and the sample size is small e.g., [30,31,32]. In more ethnically diverse postal surveys, the response rate has been around 30%, e.g., [33,34].

In the case of the FinMonik survey, the response rate was highest among women, those aged 55 years or older, widows, those with higher education, the entrepreneurs and upper level employees, those who had moved to Finland from Asia and those whose residence time in Finland was between one to four years. Furthermore, those who lived in Central Finland and Northern Savonia responded most actively. According to the previous Finnish population studies among the whole population, older age groups (55+) responded more actively than persons with lower economic status [35]. It should be noted that the non-response rates may result in participation bias. Often, those who would need more support, such as social and health services, may leave surveys unanswered. This can give an unnecessarily positive picture of the health and well-being of the target group.

The data were primarily collected online using an electronic questionnaire, but it was supplemented with a paper questionnaire, telephone interviews and home visits in the city of Espoo. This data collection method proved to be a relatively cost-effective way to obtain an extensive and representative sample. The cost benefits of online data collection are well known from previous research [36,37]. In the FinMonik survey, the electronic questionnaire was preferred by the younger age groups, by the single and the married, by those with higher education and by entrepreneurs, upper level employees and students. According to Smith et al. 2013, tertiary-educated participants are more likely to answer an online questionnaire than a paper questionnaire [38]. In the case of the FinMonik survey, it is not possibly to say whether the participants preferred a certain mode of completion because the online questionnaire was only later supplemented by the paper questionnaire and the phone interviews, and therefore, for the first five weeks of the survey, it was only possible to respond to the online questionnaire. However, this study was able to show that online data collection as a sole data collection method is not sufficient for achieving a satisfactory level of response activity. This clearly indicates that combining an online questionnaire with a paper follow-up questionnaire and phone interviews can lead to increased response activity in surveys among migrant populations.

Bhopal (2014) proposes that an ideal population survey include participants from all minority groups and provide information that is comparable across all the groups [9]. Information should be interpreted in a way that advances science, improves health status and promotes better health care. In the FinMonik survey, the study subjects were people of working age born abroad with a foreign background and currently living in Finland (PFB). Suitable comparable data for the FinMonik survey were provided by the FinSote 2018 survey of the general population: the time of data collection and data collection methods of the two studies were similar (except for telephone interviews, which were not part of the FinSote 2018 survey). The main results of the FinMonik survey have been published in a comprehensive study report (in Finnish) [39] and online [40]. The most important finding was that compared to the general population, PFBs are less likely to consider their health as good or fairly good. The findings on PFBs’ health, well-being and access to care slightly varied between both the country groups and the counties.

To ensure the comparability of the results of the FinMonik survey to those obtained among the general population, the survey questions used consisted primarily of questions that had been used in previous surveys among the general population in Finland. Previously validated translations in all the 18 languages used were not available, so two translation service providers were employed in order to ensure the quality of the translated material. The translation process was both time consuming and expensive, and still, some corrections to translations had to be made in later stages. Instead of blindly trusting translation service providers, we would recommend that all study questions and translations be scrutinized by representatives of the target group in order to ascertain that the translation products are satisfactory. Since most of the research data were collected by an online or paper questionnaire, the feedback on the quality of the translations and the comprehensibility of the questions was obtained from the study subjects. Because of the possibly poor quality of some of the translations, it is difficult to assess the reliability of the data, especially in the case of questions that had not been used in other studies or that were not included in the telephone interviews. However, the level of comparability to the FinSote 2018 study is considered to be particularly high as many of the questions in the two studies are the same. Previous studies have proposed that the translation process be carried out by a committee or a team that includes representatives from the target group as well as researchers and linguists [20,41]. Furthermore, cognitive interviews could be used to test the questionnaires. Cognitive interviewing is a method in which the questionnaire is developed together with a representative or representatives from the target population [42,43].

The FinMonik data were combined with information available in several national registers. Combining register and survey data renders conducting non-responses analyses possible and opens up a range of research questions that could be examined in further studies among different population groups as well as the general population.

## 5. Conclusions

The aim of this study was to describe the methods of the Survey on Well-Being among Foreign Born Population (FinMonik). Another aim was to present the demographics of the participants in each response format (the electronic questionnaire, the paper questionnaire and the phone interviews). Our findings suggest that combining an online questionnaire with a paper follow-up questionnaire and supplementary phone interviews can increase the response activity to a satisfactory level in migrant studies. An online questionnaire was preferred, especially by the younger age groups, the single and the married, the more highly educated, entrepreneurs, upper level employees and students. The paper form was answered particularly frequently by those aged 55–64 and widows. Although the mixed method for data collection may increase the response rate significantly, the different response methods may cause a biasing effect in responses. Further research in the field should look more closely into the methods of evaluating data quality in the context of migrant studies, in particular the potentially biasing effect of the different forms of survey, such as the electronic questionnaire, the paper questionnaire and the phone interview.

## 6. Collaboration

The FinMonik dataset is administrated by the Finnish Institute for Health and Welfare (THL), and the survey was conducted in close collaboration with the Ministry of Economic Affairs and Employment (TEM), the Ministry of Justice (OM), Statistics Finland, the Finnish Institute of Occupational Health (TTL) and the six largest cities in Finland (Helsinki, Espoo, Vantaa, Turku, Tampere and Oulu).

## Figures and Tables

**Table 1:** Overview of the survey measures and the number of questions used in questionnaire and phone interview.

Items	Sub-Items	Questionnaire	Phone Interview
Living conditions and quality of life	quality of life; psychological well-being [23]	13	6
Inclusion	contact with friends and relatives; taking part in organized activities; number of good friends living in Finland; loneliness [24]; getting help from people close to oneself, regularly helping someone in one’s household with limited functional capacity * [24]; voting; sense of belonging; trust in services	66	4
Discrimination, safety and treatment	experiences of verbal offenses, negative gestures, threat of violence, vandalism, exclusion from a group, physical abuse, sexual abuse and other threat; perceived safety	21	7
Lifestyle	leisure time physical activity; nightmares and trouble sleeping, food intake *; oral health behavior, smoking *; alcohol consumption (AUDIT-C) [24]; gambling	14	6
Health	self-rated health; limitations caused by a health problem, conditions diagnosed by a doctor, height, weight.; chronic illnesses; circumcision/female genital mutilation	15	11
Questions for women	births; abortions; miscarriages; breast and cervical cancer screenings [24]	8	-
Functional capacity and work ability	physical functional capacity, cognitive capacity, work capacity [24]	8	1
Services	healthcare appointments; access to healthcare services; social and healthcare services’ adequacy	22	9
Employment	principal employment situation; part-time work; employee/employer status; permanency of current employment; additional employments; way of finding current employment; overqualification; previous employments; job seeking	13	4
Barriers in the working life	barriers to the involvement in the working life; discrimination at workplace	19	11
Background	gender; household income * [24], adequacy of income, living conditions, access to Internet, electronic identification	16	1
Education and training	degree of education received in Finland and abroad; language skills	4	3
Immigration	Finnish citizenship; years lived in Finland; reason for migration; family in Finland and abroad	10	-

* Modified from the original.

**Table 2:** Sample size and response rate according to survey format and background information.

	All Responded *n* (%)	Electronic Questionnaire, Respondents *n* (%)	Paper Questionnaire, Respondents *n* (%)	Phone Interview, Respondents ^2^ *n* (%)	Total Sample *n* (%)
**All**	6836 (53.1)	4618 (35.9)	1878 (14.6)	340 (2.6)	12,877 (100%)
**Gender:**					
Male	3090 (50.0)	2183 (35.3)	741 (12)	166 (2.7)	6182
Female	3746 (56.0)	2435 (36.4)	1137 (17)	174 (2.6)	6695
**Age:**					
18–24 yr.	652 (48.0)	515 (37.9)	116 (8.5)	21 (1.5)	1358
25–34 yr.	1926 (52.9)	1428 (39.3)	417 (11.5)	81 (2.2)	3638
35–44 yr.	1928 (53.6)	1322 (36.8)	506 (14.1)	100 (2.8)	3595
45–54 yr.	1271 (49.8)	836 (32.8)	353 (13.8)	82 (3.2)	2551
55–64 yr.	959 (55.3)	517 (29.8)	386 (22.2)	56 (3.2)	1735
**Marital status:**					
Single or unknown	2214 (48.2)	1632 (35.6)	475 (10.4)	107 (2.3)	4589
Married or domestic partnership	3953 (57.6)	2632 (38.3)	1149 (16.7)	172 (2.5)	6865
Divorced	596 (46.3)	323 (25.1)	217 (16.9)	56 (4.4)	1285
Widowed	73 (53.3)	31 (22.6)	37 (27.0)	5 (3.6)	137
**Highest obtained education:**					
Primary level	3316 (50.3)	2227 (33.8)	937 (14.2)	152 (2.3)	6592
Secondary level	1728 (50.7)	1071 (31.4)	539 (15.8)	118 (3.5)	3408
Higher education	1792 (62.3)	1320 (45.9)	1085 (37.7)	70 (2.4)	2877
**Work Status:**					
Entrepreneur/upper level employee	1971 (57.1)	1405 (40.7)	466 (13.5)	100 (2.9)	3449
Employed	1796 (52.1)	1156 (33.5)	1651 (47.9)	95 (2.8)	3447
Student	969 (53.6)	728 (40.3)	839 (46.4)	51 (2.8)	1808
Unemplyed/retired/other	2100 (50.3)	1329 (31.8)	2073 (49.7)	104 (2.5)	4173
**Country of origin:**					
Russia or former Soviet Union	1961 (53.5)	1239 (33.8)	597 (16.3)	125 (3.4)	3668
Estonia	913 (50.4)	526 (29.0)	280 (15.5)	107 (5.9)	1811
Middle East or North Africa	1075 (55.6)	726 (37.5)	277 (14.3)	72 (3.7)	1934
Other parts of Africa	322 (46.0)	216 (30.9)	94 (13.4)	12 (1.7)	700
India, Vietnam, Thailand or other parts of Asia	1236 (58.1)	882 (41.4)	340 (16.0))	14 (0.7)	2129
EU- and EFTA -countries or North America	1018 (52.2)	797 (40.9)	215 (11.0)	6 (0.3)	1949
Other countries ^1^	311 (45.3)	232 (33.8)	75 (10.9)	4 (0.6)	686
**Lived in Finland:**					
1–4 yr.	1888 (58.5)	1445 (44.8)	407 (12.6)	36 (1.1)	3227
5–10 yr.	2276 (54.2)	1538 (36.6)	616 (14.7)	122 (2.9)	4199
10+ yr.	2672 (49.0)	1635 (30.0)	855 (15.7)	182 (3.3)	5451
**Age at migration:**					
15 yr. or younger	521 (41.8)	380 (30.5)	118 (9.5)	23 (1.8)	1247
15–19 yr.	589 (47.9)	419 (34.1)	132 (10.7)	38 (3.1)	1230
20–29 yr.	2714 (53.2)	1922 (37.7)	670 (13.1)	122 (2.4)	5104
30+ yr.	3012 (56.9)	1897 (35.8)	958 (18.1)	157 (3.0)	5296

^1^ Latin America, Yugoslavia and other countries, ^2^ A short version of the questionnaire used.

**Table 3:** Register data available for the Survey on Well-Being among Foreign Born Population (FinMonik) 2018–2019 and the FinSote National Survey of Health, Well-Being and Service Use 2018 samples.

Organization	Data
Digital and Population Data Services Agency (The Finnish Digital Agency)	Date of birth, mother tongue, service language, gender, place and type of residence, number of people in the household, country of birth, nationality, date of migration, marital status
Finnish Institute for Health and Welfare (THL)	Care Register for Health Care (inpatient and outpatient care, diagnoses, operations and other care procedures) Register of Primary Health Care Visits Cancer Registry (diagnosed cancers) Mass Screening Registry (mammography and pap smear) Medical Birth Register (childbirths, prenatal care and care during births) Register of Induced Abortions (year(s) and types of procedures) Register of Sterilizations National Infectious Diseases Register Register of Social Assistance (households receiving social assistance)
Statistics Finland	Education, occupation, socioeconomic status, income

**Table 4:** Most common countries of birth according to the country groups.

Country Group:	Country of Birth (Number of Individuals in the Sample) ^1^
Russia and former Soviet Union	Soviet Union (2747), Russia (714), Ukraine (115), Belarus (20), Kazakhstan (18), Moldova (16), Uzbekistan (13), Armenia (7)
Estonia	Estonia (1462), Soviet Union (335), Russia (9)
Middle East and North Africa	Iraq (440), Afghanistan (321), Iran (320), Turkey (284), Syria (138), Morocco (93), Pakistan (86), Sudan (59), Egypt (46), Tunisia (27), Algeria (25), Israel (23), Lebanon (21), Saudi Arabia (17), Jordan (12)
Other parts of Africa	Somalia (166), Nigeria (91), Ethiopia (72), Ghana (57), Democratic Republic of the Congo (57), Kenya (53), Cameroon (36), Gambia (25), Tanzania (18), South Africa (16), Angola (15), Eritrea (12), Senegal (11), Rwanda (10), Zambia (10), Uganda (8), Liberia (5), Côte d’Ivoire (5), Zimbabwe (5)
India, Vietnam, Thailand and other parts of Asia	Thailand (597), China (389), Vietnam (331), Philippines (162), India (142), Nepal (140), Myanmar (89), Bangladesh (80), Japan (53), Sri Lanka (40), Indonesia (24), Malaysia (19), South Korea (15), Hong Kong (11), Cambodia (11), Taiwan (10), Singapore (7)
EU- and EFTA-countries and North America	Poland (249), Germany (167), United Kingdom (162), Hungary (141), Romania (123), Bulgaria (121), United States (106), Sweden (93), Spain (89), Latvia (81), Greece (60), the Netherlands (51), Lithuania (36), the Soviet Union (36), Belgium (25), Canada (24), Norway (24), Czechoslovakia (23), Switzerland (22), Slovakia (20), Ireland (19), Austria (19), Denmark (19), Yugoslavia (18), Portugal (16), Croatia (14), Iceland (9), Czech Republic (9)
Latin America, former Yugoslavia and other countries	Yugoslavia (285), Brasil (56), Bosnia and Herzegovina (38), Cuba (27), Mexico (27), Peru (27), Albania (26), Serbia and Montenegro (26), Columbia (21), Chile (20), Australia (16), Venezuela (16), Serbia (14), Argentina (10), Dominican Republic (9), Kosovo (9), Bolivia (8), Ecuador (8), Macedonia (8), New Zealand (7), Uruguay (6)

^1^ at least five individuals in the sample.

## Data Availability

The data presented in this study can be requested from the corresponding author.
